# Diversity in global gene expression and morphology across a watercress (*Nasturtium officinale* R. Br.) germplasm collection: first steps to breeding

**DOI:** 10.1038/hortres.2015.29

**Published:** 2015-07-08

**Authors:** Adrienne C. Payne, Graham J.J. Clarkson, Steve Rothwell, Gail Taylor

**Affiliations:** 1 Centre for Biological Sciences, Institute for Life Sciences, University of Southampton, Southampton, SO17 1BJ, UK; 2 Vitacress Salads Ltd, Lower Link Farm, St Mary Bourne, Andover, Hampshire, SP11 6DB, UK

## Abstract

Watercress (*Nasturtium officinale* R. Br.) is a nutrient intense, leafy crop that is consumed raw or in soups across the globe, but for which, currently no genomic resources or breeding programme exists. Promising morphological, biochemical and functional genomic variation was identified for the first time in a newly established watercress germplasm collection, consisting of 48 watercress accessions sourced from contrasting global locations. Stem length, stem diameter and anti-oxidant (AO) potential varied across the accessions. This variation was used to identify three extreme contrasting accessions for further analysis. Variation in global gene expression was investigated using an Affymetrix *Arabidopsis* ATH1 microarray gene chip, using the commercial control (C), an accession selected for dwarf phenotype with a high AO potential (dwarfAO, called ‘Boldrewood’) and one with high AO potential alone. A set of transcripts significantly differentially expressed between these three accessions, were identified, including transcripts involved in the regulation of growth and development and those involved in secondary metabolism. In particular, when differential gene expression was compared between C and dwarfAO, the dwarfAO was characterised by increased expression of genes encoding glucosinolates, which are known precursors of phenethyl isothiocyanate, linked to the anti-carcinogenic effects well-documented in watercress. This study provides the first analysis of natural variation across the watercress genome and has identified important underpinning information for future breeding for enhanced anti-carcinogenic properties and morphology traits in this nutrient-intense crop.

## Introduction

Watercress (*Nasturtium officinale* R. Br.) is a leafy crop consumed globally, which is highly nutrient intense^1^ and contains a wide range of natural, bioactive plant compounds for which there is increasing evidence for beneficial effects on human health.^2,3^ In particular, high concentrations of glucosinolates and their hydrolysis to isothiocyanates are now known to have a role in supressing tumour growth^4,5^ and in the prevention of several other diseases including cardiovascular, neurodegeneration and diabetes.^6,7^ At the same time, global consumption of this crop is increasing. In western diets this tends to be as a salad leaf eaten raw, whilst in eastern diets, the leaves and stems of the plant are cooked and used in soup.^8^ In a world where food security is a global concern and where sustainable intensification suggests that primary production must be achieved more efficiently, the availability of food plants with high nutritional value is likely to become more important in the future.^9,10^ Despite this, limited genetic or genomic resources have been procured for watercress and there is no active breeding programme, globally. Watercress has not, to date, been fully DNA sequenced but recent advances in next generation sequencing mean that rapid progress should now be possible. Next generation sequencing opens a whole new expanse of opportunities to examine the genome at relatively low cost even in an under studied crop such as watercress.^11^ However as a first step, insight into variation in traits of interest is still required. Given this, it is important to characterise wide germplasm collections phenotypically in order to target NGS resources effectively, in addition to identifying germplasm for breeding.

Wild populations or germplasm collections often contain beneficial genetic variation that can be introgressed into breeding programmes,^12^ including for agronomic, nutritional or stress tolerance traits. Examples of introgression of such wild alleles into breeding programmes have been previously demonstrated^13^ including for increased drought and salinity tolerance which was achieved in tomatoes by introgression of genes from wild relatives.^13^ Traits such as soluble content and fruit colour have also been improved in tomatoes.^14^ Wheat, barley and rye have a limited genetic diversity and the success in breeding programmes of these cultivars is largely driven by the introgression of the alleles from wild relatives.^15^ Thus, for watercress, analysis of wild populations may identify useful material for breeding crops with improved phytonutrients, in addition to aiding the search for suitable material from which to develop genetic and genomic resources.

A cross species GeneChip, termed X species has been used for a range of species and in this study we hybridised watercress to the *Arabidopsis* chip for gene expression analysis. *Arabidopsis* was selected as like watercress it is a member of the Brassicaceae family.^16^ Several papers have reported using arrays designed for a model species to investigate the transcriptome of other species e.g. Hammond *et al.*^17^ investigated *Brassica oleracea* on the ATH1 genechip with 98.8% of the available probe sets retained, *Thlapsi caerulescens* and *Thlapsi arvense* were hybridised to the *Arabidopsis* ATH1 genechip,^18^ banana (Musa species) was hybridised to both the ATH1 and rice genechip^19^ and finally *Cardamine kokaiensis* hybridised to the ATH1 genechip.^20^

The *Arabidopsis* ATH1 chip contains 22,810 *Arabidopsis* probe sets allowing for the evaluation of almost all the genes in the *Arabidopsis* genome.^21^ The coding regions in *Arabidopsis thaliana* and across Brassicaceae are highly conserved with Cavell, Lydiate^22^ reporting an 85% sequence similarity.^23^ Also on a Brassica expressed sequence tag (EST) microarray made available from John Innes centre, 77% of the protein hits came from *A. thaliana*. This gave us confidence to proceed with using *Arabidopsis* to further watercress knowledge.

The main aim of this study was to provide underpinning data for molecular breeding by quantifying natural genetic diversity for phenotype, genotype and gene expression data (the transcriptome), in a global watercress germplasm collection held at the University of Southampton. Growth measurements were made on the collection in two contrasting environments and a broad screen for phytonutrient status was used to assess anti-oxidant (AO) potential, using FRAP.^24^ FRAP is a simple assay which assesses “AO potential” by ferric to ferrous ion reduction causing the formation of a ferrous-tripyridyltriazine complex which is measured using a spectrophotometer for colour change.^25^ Although AO potential is a generic measurement, it provides insight into the AO potential of a wide range of phytonutrients including vitamins, phenolics, sulphur-containing compounds and glucosinolates. Our analysis provides the first assessment of functional genomic and biochemical diversity in a wild watercress collection from across several continents and as such will be important for future targeted sequencing and molecular breeding for the sustainable intensification of this nutrient-intense food crop.

This study sought to identify natural variation within a watercress germplasm collection which could be taken forward for a molecular breeding programme focused on enhancing nutritional and agronomic traits.

## Materials and methods

### Growth and morphology analysis

#### Watercress germplasm collection and growing conditions

Seeds were collected from commercial growers, from a variety of locations and donated from HRI, Warwick. Seeds were germinated in Petri dishes lined with two layers of moistened filter paper with lids but not sealed and then individually transferred to peat (pH 6, mg/litre added; N-144, P-154 and K-264) in 7.5 cm pots. The pots were then randomised and placed in large trays which were maintained with 50 mm of water and checked on a regular basis. The watercress accessions were raised in a growth room with a controlled environment (CE), 25 °C day/22 °C night, 16 hours day, 06:00–22:00 with PAR varying between 120 and 180 µmol m^−2^ s^−1^ and humidity uncontrolled. After 7 weeks of growth the whole plant minus roots were snap frozen for DNA and RNA extraction. For accessions grown in the field the accessions were initially germinated in 53 × 32 cm (32 squares by 18 squares) sowing trays kept in a polytunnel located in Spetisbury (50°48′46.8″N, 2°08′47.9″W), Dorset, UK with continual misting and then transferred to a watercress bed which was gravel lined with free flowing spring water. The bed was divided into sections to ensure no mixing of accessions occurred.

### Morphological

#### Stem length, diameter and leaf number index

The length of the longest stem on each plant was recorded in centimetres and the stem diameter at 5 cm up the stem was recorded for each plant after 7 weeks. The number of fully expanded leaves (leaves larger than 2 cm × 2 cm) was recorded. The leaf number index (LNI) was then calculated by dividing the number of leaves per plant by the stem length of that plant.

### Statistical analysis

Two-way ANOVA model was applied on morphological and biochemical data to determine statistical significance:

Y=A+B+AB

where A = watercress accession and B = growth environment.

#### Anti-oxidant potential: ferric reducing ability of plasma (FRAP)

The FRAP method was based on that of Benzie and Strain^25^ but modified by Payne *et al.*^24^

### Genetic analysis

#### DNA extraction

DNA extraction was carried out using a CTAB protcol adapted from Doyle.^26^

*Hybridisation to X species chip.* DNA from the control line (C) was sent to the National Arabidopsis Stock Centre (NASC) for hybridisation to the X species ATH1 genome array.

*Xspecies chip analysis – creation of probe mask (.cdf) files.* Oligonucleotide GeneChip probes with low/no hybridisation to watercress transcripts were removed using the gDNA probe masking strategy devised by Hammond *et al.*^17^ and subsequently used by Davey *et al.*^19^ The custom .cdf file was then loaded onto the GeneSpring software for analysis of the CEL files.

*RNA extraction.* RNA was extracted from three accessions with traits of interest; control (C), high AO content and high AO content with a dwarf phenotype (dwarfAO). Accessions were grown in a CE in order to eliminate any environmental effects.

RNA was extracted using the Zymo Research (ZR) kit (Zymo Research, Irvine, CA, USA) following manufacturers guidelines.

*Microarray analysis and hierarchial clustering.* After selection of perfect-match probes which hybridised efficiently to the gDNA the perfect match probes were used for interpreting the GeneChip array in which RNA from *Nasturtium officinale* was hybridised. Examination of gene expression was conducted using the Agilent Genespring Gx Analysis Workbench version 7.3.

Microarray analysis was performed according to the Agilent GeneSpring GX version 7.3 analysis workbench manual and Mapman 3.1 instruction manual. Microarray analysis allows the expression of thousands of genes to be investigated simultaneously.^27^ The .cdf file (filtered from the raw cel.file) created from the X species analysis was used for the gene expression analysis. Robust multiarray averaging (RMA) normalisation was used. Statistical analysis to identify differentially expressed genes was performed with ANOVA using a *p* cut-off of 0.05. Multi-experiment Viewer (TMeV) was used to perform hierarchical clustering, using the standard Pearsons and a distance of 0.28.

*cDNA synthesis and real-time PCR.* cDNA synthesis was performed with the ImPromII Reverse Transcription kit, Promega (Southampton, UK) and manufacturers guidelines followed. Real-time PCR was performed using At1g65060 (encodes an isoform of 4-coumarate:CoA ligase (4CL) and At4g35000 (encodes a microsomal ascorbate peroxidase APX3). A 1/4 dilution of the cDNA for each sample was used, along with the primer mix and 2X SYBR master mix (Finnzymes, Thermo Scientific, Loughborough, UK). Chromo4 real-time PCR machine (Biorad) was used with the following programme: 95 °C for 10 minutes, 95 °C for 10 seconds, 60 °C for 15 seconds, 72 °C for 15 seconds, 75 °C for 1 second, plate read, repeat from the second step 39 more times, 72 °C for 5 minutes, melting curve from 55 °C to 95 °C, read every 0.2 °C, hold for 1 second and finally 75 °C for 5 minutes. Gene expression calculations to confirm microarray results were performed using the methods of Pfaffl.^28^

*Mapman analysis.* Mapman is a user-driven tool which displays large data sets and displays gene expression experiments on diagrams of metabolic pathways or other processes. Gene expression data were obtained from microarray analysis and the following calculation performed in order to determine differences in expression which were illustrated in Mapman.

Expression = log (gene expression of accession of interest/gene expression of control accession)

## Results

### Morphological and biochemical variation in a watercress germplasm collection

**Figure 1:**
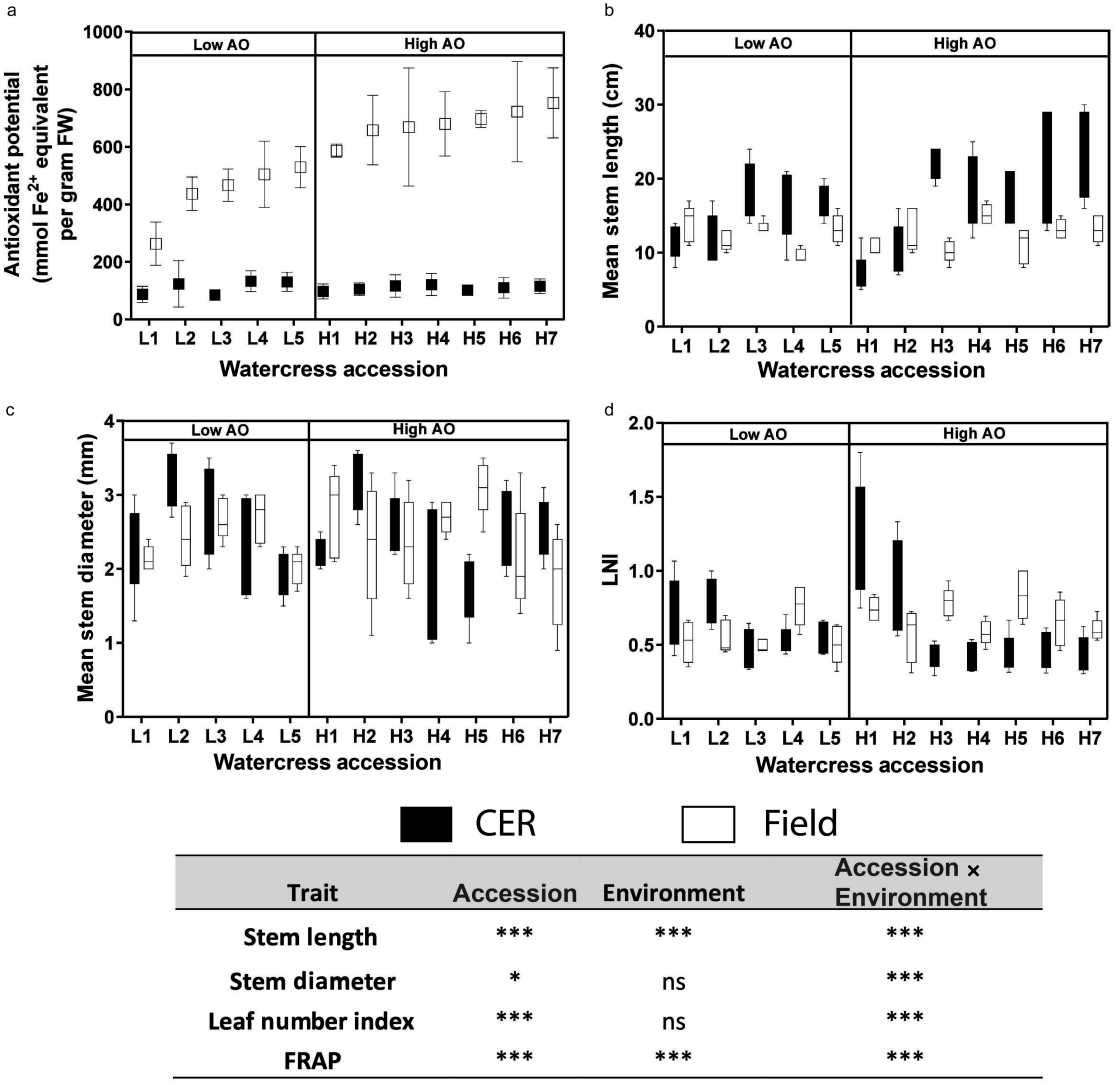
(a) anti‐oxidant potential (b) mean stem length (c) mean stem diameter (d) leaf number index of the watercress accessions held within the germplasm collection. L1‐5 represent watercress accessions which had a low anti‐oxidant potential (<600 mmol Fe^2+^ per gram FW) whilst H1‐7 represent accessions with a high anti‐oxidant potential (>600 mmol Fe^2+^ per gram FW). Asterisks identify significance from two way ANOVA analysis *p < 0.05, **p < 0.01, ***p < 0.001. CE refers to the growth of the plants in a controlled environment in which environmental conditions are set to ensure minimum fluctuations and therefore any morphological or biochemical differences can be attributed to genes and not the environment.

AO potential from FRAP differed significantly across the watercress accessions when grown in a CE and also when grown in the field (Figure 1a). There was a dramatic increase in AO potential for field grown as compared to CE-grown watercress where the control accession increased from 130 mmol Fe^2+^ equivalent per gram fresh weight in a CE to 529 mmol Fe^2+^ equivalent per gram fresh weight in the field. There was a significant difference between the watercress accessions, environment and an interaction between accession and environment (Table 1). The different watercress accessions did not rank the same in the CE and field and Spearman’s rho revealed a non-significant relationship, *r_s_*(34) = 0.019, *p* = 0.915.

Stem length differed significantly across the watercress accessions irrespective of growth environment and varied by 74% and 44% in CE and field respectively (Figure 1b). When the CE grown watercress was compared to the field there was a distinct difference. The CE grown accessions had a longer stems compared to field grown accessions. There was a significant difference between the watercress accessions, environment and an interaction between accession and environment (Table 1). The different watercress accessions did not rank the same in the CE and field and Spearman’s rho revealed a statistically non-significant relationship, *r_s_*(58) = 0.075, *p* = 0.569.

Stem diameter also differed significantly across the watercress accession lines in both CE and field (Figure 1c). The mean stem diameter was generally higher for CE grown watercress compared to accessions grown in the field. Stem diameter differed significantly between watercress accessions but did not significantly differ between environments however there was a significant interaction between accession and environment (Table 1). The different watercress accessions do not rank the same in the CE and field and Spearman’s rho revealed a statistically non-significant relationship, *r_s_*(58) = −0.179, *p* = 0.171.

LNI was calculated as leaf number per unit stem length. LNI was highest for field grown watercress, possibly indicating a response to competition where swards of watercress were grown together (Figure 1d). LNI differed significantly between watercress accessions but did not significantly differ between environments however there was a significant interaction between accession and environment (Table 1). The different watercress accessions do not rank the same in the CE and field and Spearman’s rho revealed a statistically non-significant relationship, *r_s_*(58) = −0.005, *p* = 0.969.

In summary, phenotypic and biochemical measurements revealed significant variation between the watercress accessions which led to an examination of gene expression.

### Assessing global gene expression in a watercress germplasm collection

*X species analysis*^17,19^ Using .cdf masking the hybridisation to the *Arabidopsis* ATH-1 gene chip was very high with a minimum intensity being set at 16 000 in order to obtain no probe sets or probe pairs being retained. In order to determine the threshold intensity only the data from 0 to 2000 were plotted so the greatest distance between the probe-set and probe-pair could be identified. It appears that the threshold intensity was 80. Investigation into any variation in gene expression between the three selected accessions (C, AO and dwarfAO) could then be explored.

### Venn diagrams; visualisation of commonalities between gene lists

**Figure 2:**
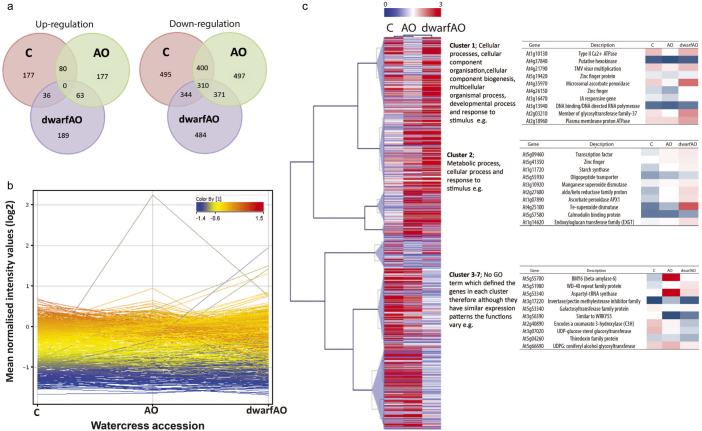
(a) Up‐ and down‐regulated gene expression for control (C), high anti‐oxidant (AO) and dwarf, high AO accession (dwarfAO). (b) Mean expression ratio for total gene expression of C, AO and dwarfAO, where green represents down‐regulation, pink represents an up‐regulation of genes in accession C when RMA normalisation to the median was performed. This provides clear visualisation of differing expression of specific genes between three watercress accessions. For example, a gene which is down‐regulated in C will have a green line despite being up‐ or down‐regulated in AO and dwarfAO due to the colour of the line being dependent on expression in C. This aids the visualisation of the change in expression without having to trace the accession back to C. (c) Hierarchical clustering of changes in transcript abundance for C and AO and dwarfAO, performed using TMeV software from normalised expression ratio data. Lilac triangles represent seven different clusters, colour scale indicates signal normalised ratios (red = up‐regulated genes, blue = down‐regulated genes). Only genes with ANOVA significance *p* ≤ 0.05 are shown.

Figure 2a explores the commonalities between gene lists of significantly different expression between the three accessions of interest, i.e. how many genes in common were up- or down-regulated between these three accessions. This would give an indication of the similarity of these three accessions to each other (control C; high AO; AO and dwarf high AO; dwarfAO). The up-regulation of genes was filtered on expression above 0 and the down-regulation of genes filtered on expression below 0 after log_2_ had been applied. The examination of up- and down-regulated genes was conducted on lines C, AO and dwarfAO. Accession C and AO had an up-regulation of 80 genes in common whilst only 36 genes were up-regulated in common between accession C and dwarfAO, between accession AO and dwarfAO 63 genes were in common. dwarfAO had the highest number of genes up-regulated with 189 genes in total whilst AO and C had 177 genes. Interestingly there were 0 genes in common for all three accessions (Figure 2a). It is evident that the accessions C, AO and dwarfAO differ at the level of the transcriptome as illustrated in the gene expression output from microarray analysis in Figure 2b, dwarfAO demonstrating the most distinct difference.

Again for down-regulation accession C and AO had the highest number of genes in common down-regulated, 400 genes whilst C and dwarfAO had the lowest number of genes in common down-regulated, 344 genes. AO had the overall highest number of down-regulated genes with a total of 497 whilst C has a total of 495 genes down-regulated and dwarfAO 484 genes down-regulated. There were 310 genes in common for all three accessions (Figure 2a).

It is important to note that it appears that C and dwarfAO have the fewest common genes up- and down-regulated. Interestingly there was a smaller difference between genes which are commonly up- and down-regulated between AO and dwarfAO and also C and AO.

### AgriGO – biological processes, cellular component and molecular function

Gene expression data were uploaded into AgriGO (Figure S1). dwarfAO tended to give the highest Z score (statistical value in PAGE) in biological processes in particular developmental process and response to stimulus. Also in terms of molecular function and cellular components dwarfAO has the highest Z score. It is evident that there is different control over various biological processes, molecular functions and cellular components across these three contrasting accessions.

### Multi-experiment Viewer (TMeV)^29^; gene clustering

There were dramatic differences in gene expression between the three key accessions of interest (control, high AO content and high AO content with dwarf phenotype) (Figure 2b). Gene expression from microarray analysis was confirmed by real-time PCR (Figure S2). The list of genes which changed significantly between the watercress accessions were then uploaded to TMeV^29^ to investigate clustering of these genes. It is evident that C and AO were more closely related with respect to gene expression than with dwarfAO (Figure 2c). This could explain some biochemical and phenotypic observations previously recorded. From the output of TMeV, seven gene clusters were revealed with two of these with significant GO term categories, i.e. enough similarity to classify the cluster with overall functions. In order to reveal the function of the genes in each of the clusters the data were uploaded to AgriGO.^30^ Clusters 1 and 2 had significant GO term categories where cluster 1 was mainly associated with cellular processes, cellular component organisation, cellular component biogenesis, multicellular organismal process, developmental process and response to stimulus whilst those in 2 were associated with metabolic process, cellular process and response to stimulus. Clusters 3–7 had no GO term which defined the genes in each cluster therefore although they have similar expression patterns the functions vary.

### Mapman – visualisation of metabolic and cellular processes

**Figure 3:**
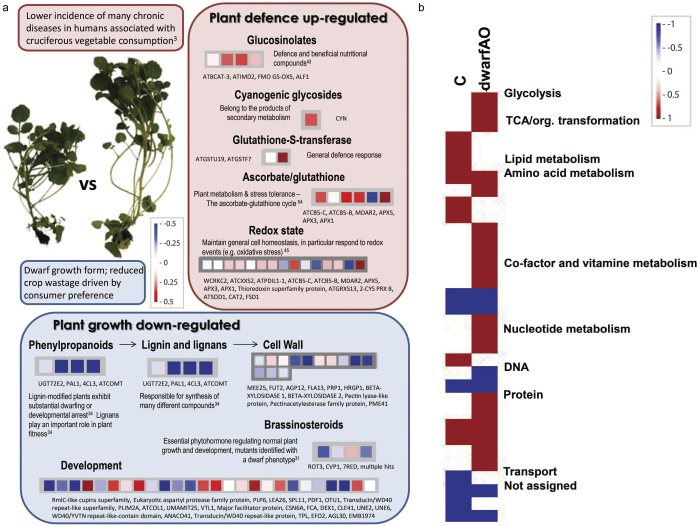
(a) Transcriptomic differences between a novel dwarf watercress accession (dwarfAO) and the commercial (C) accession, where an up- or down- regulations, red and blue squares respectively is shown in dwarfAO compared to C. Mapman output illustrating down-regulation of genes in bins relating to plant growth in dwarfAO/C and an up-regulation of genes in the bins relating to plant defence in watercress accession dwarfAO/C. Superscript numbers denote Refs. 43, 54, 45, 34, 31, 3. (b) Overview of significantly different functional categories after microarray analysis. Colour scale represents fold changes, blue down-regulation and red up-regulation. Wilcoxon rank-sum test was applied to all three comparisons performed in PageMan. Clear visual representation of how the functional ontologies differ between the two watercress accessions.

Overall there was a clear differentiation in gene expression in the dwarf phenotype in which plant defence was up-regulated whilst plant growth was down-regulated (Figure 3a). This is clearly identified by examining bins in relation to plant growth and defence. dwarfAO exhibited a down-regulation of genes involved in the control of plant growth (brassinosteroids, phenylpropanoids, lignin) and an up-regulation of genes in plant defence (glucosinolates, cyanogenic glycosides, glutathione-S-transferase and ascorbate/glutathione). Gibberellins and brassinosteroids are plant hormones which are required for growth and development. Commonly associated with a dwarf phenotype is a reduction in either of these hormones, plants which are unable to perceive brassinosteroids exhibit typical phenotypes which include the dwarf phenotype.^31^ The brassinosteroids have been reported to be involved in organ growth, cell expansion, cell division and responses to abiotic and biotic stresses.^32^ Gene expression analysis from Mapman reveals a down-regulation in the bin representing brassinosteroids for the following genes; Rotundifolia3 (at4g36380), Sterol methyltransferase2 (at1g20330), 7RED (at1g50430) and multiple hits which correspond to brassinosteroid metabolism. For example, *ROTUNDIFOLIA3* encodes an enzyme to catalyse C-23 hydroxylation of several brassinosteroids which is in fact two steps shorter than the conventional brassionsteroid pathway,^32,33^ this gene is down-regulated in dwarfAO.

Also any deviation in lignin biosynthesis, whether that is in terms of lignin modification, can result in a dwarf phenotype or developmental arrest.^34^ Consistently down-regulated in dwarfAO is phenylalanine ammonia-lyase (PAL) (at2g37040) along with 4-coumarate-CoA ligase (at1g65060), UDPG:coniferyl alcohol glucosyltransferase (at5g66690) and O-methyltransferase1 (at5g54160), involved in the phenylpropanoid pathway and lignin biosynthesis.^34,35,36,37,38^ PAL catalyses the first step in the phenylpropanoid pathway which involves the deamination of Phe to cinnamic acid.^39^ A study by Huang *et al.*^39^ reported a stunted and sterile *Arabidopsis* with the quadruple knockout of the four PAL genes, also the levels of lignin were greatly reduced. Genes in the bin regarding the cell wall were also down-regulated. Monolignol biosynthesis mutants have dwarf phenotypes as many different disruptions in lignin biosynthesis may result in a dwarfs, however further work for confirmation is required.^34^ Within the watercress accessions a down-regulation of lignin is evident in dwarfAO and also a high proportion of genes are down-regulated in terms of the phenylpropanoid pathway. There is a high proportion of down-regulation in the bin associated with development which may be a result of a variety of down-regulation in terms of brassinosteroids, lignin and the phenylpropanoid pathway resulting in the dwarf phenotype.

Perhaps more importantly here, is the initial step in the biosynthesis of glucosinolate – the key chemical constituents contributing to the anti-cancer properties in brassica crops including watercress and broccoli.^40,41,42^ Glucosinolate biosynthesis was up-regulated in dwarfAO confirming the likely improved beneficial bioactive compounds for humans in this watercress accession.^43^ Glucosinolates up-regulated include 3-isopropylmalate dehydrogenase (at1g80560) and a flavin-containing monooxygenase family protein (at1g12140). Ascorbate was also up-regulated which scavenges hydrogen peroxide, and is an important AO system of vital importance in combating oxidative stress, alongside an up-regulation of ascorbate peroxidise (at1g07890). Other genes which were up-regulated in the bin responsible for genes encoding ascorbate/glutathione include cytochrome b5 family protein #1 (at2g46650) monodehydroascorbate reductase (at5g03630), cytochrome B5 isoform (at2g32720), ascorbate peroxidase 5 (at4g35970) and ascorbate peroxidase 3 (at4g35000). Glutathione is of up-most importance to plants as plants are unable to survive without glutathione. It plays a function in plant development which cannot be performed by any other thiols or AOs.^44^ Glutathione also plays a role in prevention of oxidative stress. Redox status takes into account all the different redox compounds including ascorbate, glutathione, NAD(P)H and proteins from the thioredoxin,^45^ two of which have been mentioned. Genes in the bin regarding redox status are up-regulated in order to maintain a general redox homeostasis thereby preventing oxidative stress. The down-regulation of many genes associated with plant growth and the up-regulation of genes associated with plant defence may contribute to an understanding in the phenotype and improved nutritional properties in the dwarfAO.

### Pageman – visual representation of functional ontologies

Gene expression data were uploaded to Pageman (Figure 3b). It is apparent that accession C and dwarfAO differ in the significant functional ontologies. dwarfAO has a significant up-regulation of the following functional ontologies; glycolysis, TCA/org. transformation, co-factor and vitamine metabolism, nucleotide metabolism and DNA whilst accession C lipid metabolism and transport. Functional ontologies which are significant for both accessions include amino acid metabolism and non-assigned function. This reinforces the output of the TMeV which was used to investigate clustering of genes. From this programme, it was evident that C and AO were more closely related in respect to gene expression patterns than dwarfAO. This may also explain the distinctive dwarf phenotype and high AO potential of dwarfAO compared to C and AO. Also when the gene expression analysis was examined using Venn diagrams for C and dwarfAO it was clear that there were fewer genes in common which were up- or down-regulated.

## Discussion

The watercress germplasm collection investigated here represents an important repository of genetic diversity for future breeding effort in this species. Currently, no active breeding programme exists for watercress, globally, which is surprising given the important nutrient intensity of this crop relative to others.^1^ This study has taken the important first steps in revealing the potential of this crop. Introgression of alleles from wild relatives of crop plants is recognised as a valuable route for yield and quality improvements.^46^ Tanksley and McCouch^47^ highlight the need for germplasm collections to unlock valuable genetic information from wild relatives. Plant breeders wish to optimise the use of natural genetic variation by bringing together favourable alleles (such as maximising yield or increased resistance to stress) in one genotype^48^ and this will continue to play a vital role in agriculture and has contributed to increased crop productivity.^49^ Watercress undoubtedly has a large number of nutritional characteristics, which have yet to be introgressed into directional breeding. This study has identified watercress accessions with favourable traits both nutritionally and agronomically which can contribute to breeding programmes.

Initial phenotypic measurements included stem length, stem diameter and LNI. These showed promising variation within the collection along with biochemical analysis (FRAP for AOs). Germplasm collections offer a valuable source of genetic variation,^50^ of particular interest here was variation in stem length alongside beneficial nutritional compounds that contribute to plant defence and disease-preventing phytonutrients.^42^ Glucosinolates are sulphur containing compounds and upon breakdown form products which have beneficial e.g. anti-cancer properties.^40^ It is the characteristic peppery taste of watercress; other *Brassicas* also have distinct flavours, which contribute to cancer prevention.^40,42^ A breakdown product of glucosinolates which is vital to the beneficial anti-cancer properties of watercress is phenethyl isothiocyanate (PEITC, glucosinolate derivative). PEITC can inhibit cancer development by preventing carcinogen activation by; inhibiting phase 1 enzymes,^41^ increasing activity of detoxifying/AO phase II enzymes,^41^ inhibiting uncontrolled growth of cancer cells by inhibiting cell cycle progression,^51^ inducing apoptosis^52^ and anti-angiogenesis which prevents the formation of new blood vessels to a tumour.^53^ As stated by Cartea and Velasco,^40^ variation in glucosinolates can be attributed to both genetics and environment and here we reveal both a genetic and an environmental effect of glucosinolate metabolism with very clear differences AO potential revealed in the contrasting growth environments. The established germplasm collection is likely to contain valuable variability in the beneficial compounds, glucosinolates.

Transcriptome analysis of three accessions; C, AO and dwarfAO revealed variation in gene expression. In particular the expression of an ascorbate peroxidase defending against hydrogen peroxide^54^ and an isoform of 4-coumarate:CoA ligase (4CL) involved in the final steps of the phenylpropanoid pathway.^55^ There was an intriguing level of variation of gene expression between the three different watercress accessions, in particular between C and dwarfAO. Gene expression analysis indicated a high investment in defence chemistry by dwarfAO. For successful growth, plants are required to allocate resources between numerous processes^56^ and the growth-differentiation balance hypothesis predicts the allocation of resources.^57^ It is of great importance that the plant grows quickly in order to compete and also maintain defences in order to survive attack.^58^ dwarfAO is a valuable target for breeding as it appears to partition more resources into defence compared to growth, it has a dwarf, small leaved phenotype and a raised concentration of glucosinolates suggesting better disease-prevention phytonutrition.

This study has demonstrated the ability to examine the expression of thousands of genes and detect differences in gene expression using microarray analysis. Further steps can be initiated for a molecular breeding programme based on the vast array of genetic information now available from this study.

In conclusion this research provides the first analysis of the watercress transcriptome and has opened up a vast array of gene expression data for this crop; indeed, it has also highlighted genes of interest. By utilising a collection with considerable natural genetic diversity, we have also quantified significant phenotypic and biochemical differences related to yield, morphology and biochemical traits underpinning the health benefits of watercress consumption. This valuable pre-breeding data can now be extended to develop a targeted molecular breeding programme in watercress. Initial investigation into the watercress germplasm collection has provided exciting results which act as a valuable platform in which to progress further in terms of breeding for an enhanced cultivar. Current research has used the information here to develop new crosses, an RNASeq transcriptomic data set (Voutsina personal communication) and molecular genetic map of watercress interest. The impact of commercial breeding has resulted in a very narrow genetic basis for many crops, fortunately this study has revealed this may not be the case for watercress.

## Figures and Tables

**Table 1:** Statistical significance of traits of interest, shading represents significant results

Trait	Accession	Environment	Accession × Environment
Stem length	*F*_(11,96)_ = 9.05, *p* < 0.001	*F*_(1,96)_ = 28.16, *p* < 0.001	*F*_(11,96)_ = 7.23, *p* < 0.001
Stem diameter	*F*_(11,96)_ = 2.35, *p* < 0.05	*F*_(1,96)_ = 0.02, *p* > 0.05	*F*_(11,96)_ = 4.33, *p* < 0.001
Leaf number index	*F*_(11,96)_ = 7.15, *p* < 0.001	*F*_(1,96)_ = 0.41, *p* > 0.05	*F*_(11,96)_ = 7.58, *p* < 0.001
FRAP	*F*_(11,71)_ = 8.26, *p* < 0.001	*F*_(1,71)_ = 966.60, *p* < 0.001	*F*_(11,71)_ = 7.28, *p* < 0.001

## Notes

We confirm we have no conflict of interests.
