# Supplementary material for: Diversity in global gene expression and morphology across a watercress (Nasturtium officinale R. Br.) germplasm collection: first steps to breeding
Source: Hortic Res. 2015 Jul 8;2:15029–. doi: 10.1038/hortres.2015.29 (PMC4591680; doi:10.1038/hortres.2015.29)
Supplement: Supplementary Figures [file hortres201529-s1.pdf]

SUPPLEMENTARY MATERIAL

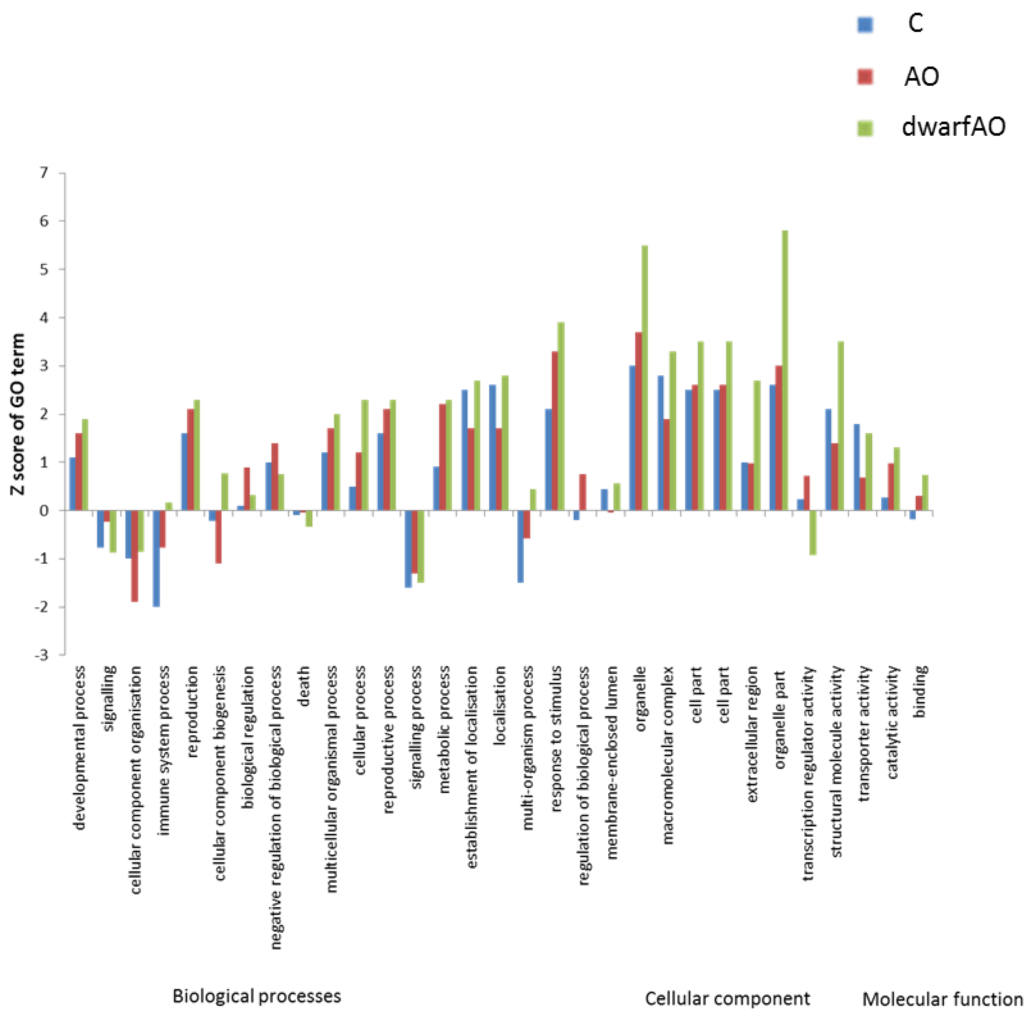

Figure S1: AgriGO output illustrating the differences in functional groups between C, AO and dwarfAO gene expression data.

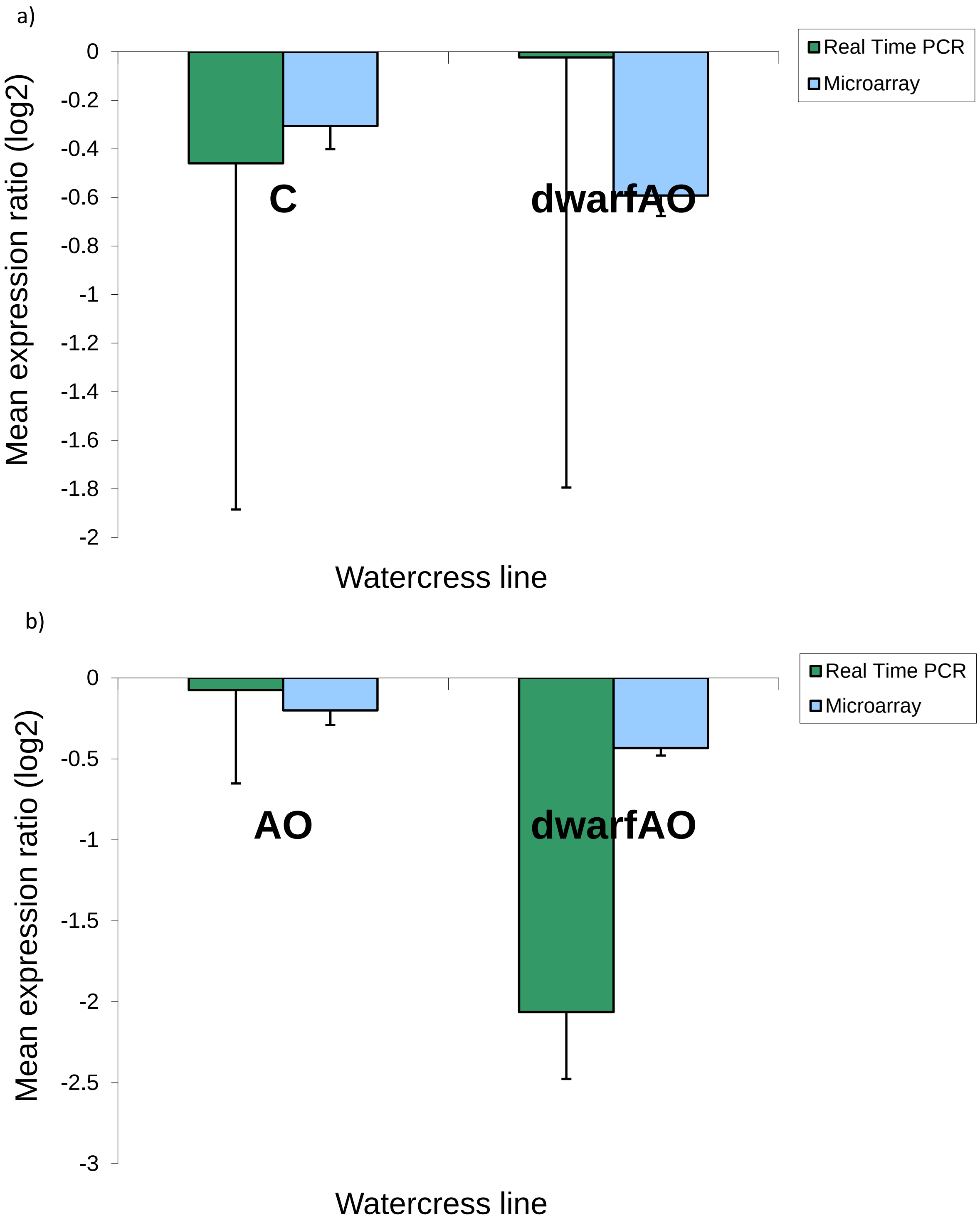

Figure S2: Real time PCR confirmation of microarray gene expression data for a) At4g35000 (encodes a microsomal ascorbate peroxidase APX3) b) At1g65060 (encodes an isoform of 4-coumarate:CoA ligase (4CL))
